# Using CCA-Fused Cepstral Features in a Deep Learning-Based Cry Diagnostic System for Detecting an Ensemble of Pathologies in Newborns

**DOI:** 10.3390/diagnostics13050879

**Published:** 2023-02-24

**Authors:** Zahra Khalilzad, Chakib Tadj

**Affiliations:** Department of Electrical Engineering, École de Technologie Supérieur, Université du Québec, Montreal, QC H3C 1K3, Canada

**Keywords:** newborn cry, Gammatone Frequency Cepstral Coefficients, Support Vector Machine, Long Short-term Memory, Hyperparameter optimization, feature fusion

## Abstract

Crying is one of the means of communication for a newborn. Newborn cry signals convey precious information about the newborn’s health condition and their emotions. In this study, cry signals of healthy and pathologic newborns were analyzed for the purpose of developing an automatic, non-invasive, and comprehensive Newborn Cry Diagnostic System (NCDS) that identifies pathologic newborns from healthy infants. For this purpose, Mel-frequency Cepstral Coefficients (MFCC) and Gammatone Frequency Cepstral Coefficients (GFCC) were extracted as features. These feature sets were also combined and fused through Canonical Correlation Analysis (CCA), which provides a novel manipulation of the features that have not yet been explored in the literature on NCDS designs, to the best of our knowledge. All the mentioned feature sets were fed to the Support Vector Machine (SVM) and Long Short-term Memory (LSTM). Furthermore, two Hyperparameter optimization methods, Bayesian and grid search, were examined to enhance the system’s performance. The performance of our proposed NCDS was evaluated with two different datasets of inspiratory and expiratory cries. The CCA fusion feature set using the LSTM classifier accomplished the best F-score in the study, with 99.86% for the inspiratory cry dataset. The best F-score regarding the expiratory cry dataset, 99.44%, belonged to the GFCC feature set employing the LSTM classifier. These experiments suggest the high potential and value of using the newborn cry signals in the detection of pathologies. The framework proposed in this study can be implemented as an early diagnostic tool for clinical studies and help in the identification of pathologic newborns.

## 1. Introduction

In 2019, 6700 neonatal deaths occurred every day, and around 75% of these deaths occurred within the first 7 days after birth; this highlights the significance of expeditious diagnosis during the first few days of any neonate’s life. Several pathologies associated with a neonate’s mortality require invasive clinical tests and a high vigilance. Unfortunately, the regions that suffer the most from high newborn mortality rates are those deficient in the number of skilled health professionals. The World Health Organization (WHO) states that two-thirds of newborn deaths could be prevented if diagnosis and treatments took place before the second week of an infant’s life. Furthermore, in most cases of pathological studies, if the treatment is initiated expeditiously, the infant may completely heal if given the right treatments [1].

As early as the 19th century, the cry of neonates was recognized as a cue in identifying morbidity [2]. The acoustic characteristics of a cry may vary due to various factors such as air pressure, tension, length, thickness, and shape of the vocal cords and resonators [3]. Experienced parents and caregivers may distinguish types of cries only by listening; however, even trained nurses could only reach an accuracy of around 33% by relying on their auditory system [4]. Healthy newborns have a fundamental frequency of 400–600 Hz, with an average of 450 Hz [5]; they also show a decreasing or increasing–decreasing melody shape with super imposed harmonics, and an average duration of 1–1.5 s [6]. The cries of babies suffering from a specific pathology are associated with low punctuation; they reflect high irritability and the physiological persistency is low [7]. Some of the features and attributes in infant cry signals can seldom be observed in healthy infants, though are commonly seen in pathologic ones [8]. For example, hypothyroidism could result in low-pitched cries, a lower number of shifts, and a frequent observance of the glottal roll at the end of phonation. Cries marked with hypothyroidism have been marked as hoarse [9]. This acoustic structure has enabled us to develop a Newborn Cry Diagnostic System (NCDS) and take a deeper look into the health status of neonates.

The study of newborn cry signals unveiled that they bear abundant helpful information about the neonate’s health conditions. Extensive research in this area has demanded an automatic approach and accurate analysis of the cry spectrographs; hence, newborn cry analysis systems were designed to overcome this challenge [10,11,12,13,14,15]. The study of newborn cry signals has multiple goals.

There are many interesting publications in the literature that analyze cry signals from aspects other than those used in this study. These studies range from the identification of the reason for crying, e.g., hunger, pain or boredom [16,17,18]; emotion detection [19]; detecting the cry in Neonatal Intensive Care Units (NICUs) and in surveillance systems [20,21]; segmenting the cry signal into its episodes [22,23]; diagnosis of specific pathologies [24] or general identification of a pathologic infant [25,26,27,28], as well as studying how each factor would affect the cry characteristics. Some of these works have explored the roles of pain intensity [29,30,31], gender [32], gestational age [33], and other similar factors in cry signals. This study focuses on a different type of application, which is diagnosing pathologies in newborns based on their cry signal. What this study tried to achieve was to exploit features that could reflect the alterations in the cry signal only as a result of being unhealthy and independent of other factors. We expected these features (and their fusion) to represent attributes in the cry signals that were not obvious in simple observations of spectrograms, and also were not affected by changes in etiological factors across newborns and the emotional state of the newborns.

Every NCDS comprises three principal stages: pre-processing, feature extraction, and classification. In the pre-processing stage, the cry signal is pre-emphasized and framed; the pauses and silences are removed, filtered, and segmented to be ready for feature extraction. Following pre-processing is the feature extraction step. The features that are capable of discriminating the healthy cry signals from the pathologic ones are exploited in this stage. These features pass through dimensionality reduction techniques and are then fed as inputs into the classifier in the last stage of the NCDS. Finally, the class labels, which were predicted by the classifier, constitute the result.

The prominent features in the analysis of newborn cry signals include the Mel-frequency Cepstral Coefficients (MFCC), owing to their good performance in the diagnostic studies of cries. MFCCs are often employed as the baseline in many experiments concerning the neonate cry. The MFCC features aid the detection of multiple diseases, such as hypothyroidism, asphyxia [34,35], hyperbilirubinemia [28], respiratory distress syndrome [10], sepsis [24,36], and cleft palate [37].

Gammatone Frequency features (GFCC) have been employed for the purpose of emotion recognition in the study of newborn cry signals [19], where they have outperformed MFCCs. GFCCs have a wide range of applications in acoustic scene classification problems, the recognition of emotions in adult speech [38], and speaker identification [39]. GFCCs were also employed in recent research identifying septic newborns from those diagnosed with RDS based on their cry, which proved to be successful [40]. Among the machine learning architectures used in infant cry analysis, Support Vector Machines (SVM) is one of the most prevalent approaches. A diversity of features such as temporal, prosodic, and cepstral have functioned successfully with SVMs [41,42,43]. Onu et al. [44] concluded that SVMs have a practical design for limited samples and data with high dimensionality, and are the most suitable for the study of asphyxiated neonates. Another classification approach employed in this work was the Long Short-term Memory (LSTM) neural network. LSTMs have been successfully paired with MFCC, GFCC, and their fusions; they showed promising performance in emotion and gender recognition applications [45,46]. However, their application has been limited in NCDS designs thus far [47]. LSTMs are one of the best choices when it comes to sequential data, such as audio signals. Nevertheless, like any other deep learning framework, LSTMs encounter the challenge of fine-tuning hyperparameters (HP) [48,49]. HP tuning can enhance the performance of a Neural Network (NN) from medium to state-of-the-art. Although many researchers emphasized the vital role of Hyperparameter Optimization (HPO) in NN architectures, only a few works have been published that suggest which and how many HPs should be optimized [50,51,52].

This study aimed to develop a comprehensive NCDS to distinguish between healthy and morbid infants as an early alert to medical staff and the guardians of the newborn. In order to obtain a comprehensive NCDS, the cry signals were analyzed regardless of cry stimulus, region, and gender. The proposed NCDS utilized both expiratory and inspiratory cry data sets. In this regard, the priority of this work was to study the role of acoustic features of the GFCC and MFCC in assessing the acoustic structure of the cry signals. Additionally, the GFCC and MFCC feature sets were combined by means of conventional and fusion methods. To the best of the authors’ knowledge, this is the first time that the Canonical Convolution Analysis (CCA) fusion of the employed feature sets has been introduced to the assessment of pathologic newborn cries. Furthermore, the discussed challenges are addressed for both classification methods through two HPO schemes, where both classifiers have been fine-tuned using the grid search and Bayesian Hyperparameter Optimization (BHPO) methods. The proposed frameworks were evaluated by several measures and the results for each one expounded and compared extensively.

This study was proposed to address multiple the challenges and shortcomings of previous studies, as represented in Table A1. A majority of the NCDS designs focus on studying a certain pathology group, whereas the aim of our work is to design a comprehensive alert system to notify the guardians of the newborn and the health professionals that the infant should undergo more screening tests, as there is a high potential it might be diagnosed with one or more pathologies from the ensemble of pathologies. Furthermore, the highest infant mortality rates are unfortunately associated with lower-income countries, where the proper screening equipment is inadequate and not available to many newborns [53]. This calls for the design of a non-complex, efficient NCDS that can perform early diagnosis so that the newborns are examined for an ensemble of pathologies and it can be determined if they are at risk of being unhealthy. As can be seen from Table A1, the studies of newborn cries, undertaken for the purpose of differentiating between healthy and pathological infants, were either performed with a less inclusive set of pathologies or included less details on how HPO would assess enhancing the NCDS design.

There are an ever-growing number of designs that trade complexity for performance; however, this study proposes that employing proper feature fusion and HPO techniques could improve an NCDS from a moderate to a highly desirable state, where all the evaluation measures are relatively high and presented. The former studies present fewer measures for the evaluation; as an example, there are a very limited number of studies that have investigated the MCC measure. Table A1 also shows that the use of HPO and fusion methods in the study of pathological newborn cry signals is inadequate. As an example, most of the presented studies employed the SVM classifier. However, the resulting values are far lower than those presented in this study (the same explanation applies to the LSTM classifier, where the results are around 10% lower without the use of HPO methods). The aim of this study is to highlight the effects and importance of HPO and fusion methods in all NCDS designs, by explaining run-times and comparing the results before and after fusion and employing HPO. The role of feature fusion and HP tuning could be crucial and shed light on many further applications that employ various modalities for developing a comprehensive system; thus, we tried to provide a detail-oriented study of how each step of the NCDS design contributed to enhancing or decrementing the final results, which distinguishes our study from other research in the field of cry-based diagnostic systems.

## 2. Methods and Participants

### 2.1. Cry Dataset and Participants

The first challenge in sketching a pathological study is the acquisition and collection of data. It is important to note that the priority is obtaining the consent of newborns’ guardians to record the cry signal and then achieving their consent to include that cry signal in the database. Furthermore, obtaining the ethical approvals to add samples to a database is an arduous and toilsome process that might even lead to losing some of the acquired data.

The collection of data was accomplished by collaboration between Al-Sahel and Al-Raee hospitals in Lebanon and Saint Justine Hospital of Montreal, QC, Canada. All the signals have been recorded in NICUs or maternity rooms (public and private) in the hospital environment. The cry of the newborns in our dataset was initiated due to multiple reasons such as hunger, fear, and wet diapers [54]. The reason for crying was resolved with the help of medical staff and newborn’s caregivers regarding the conditions resulting the cry.

Cry recordings ranged from 1 to 4 min including silence, hiccups, inspiration cries, expiration cries, and background noise. They were collected using a digital 2-channel Olympus handheld recorder with a 16-bit resolution and 44,100 Hz sampling frequency. The recorder was placed in the 10-to-30 cm vicinage of the newborn’s mouth with no special consideration in the acquisition process. The mean recording length is 90 s and there were up to 5 recordings from each newborn. Therefore, unwanted information such as chatter in the surrounding space, noises, instrument beeps, and cries of other newborns accompanied the signals, which makes our dataset a real corpus capable of solving the challenge of comprehensiveness. Moreover, the newborns included in our dataset represent different races, origins, genders, and weights. A summary of this dataset is represented in Table 1.

Newborns do not have any control over their vocalization before 3 months of age (more accurately, 53 days) [55]. The genesis of vocalizations in advance of this age is merely affected by biological rhythms. Moreover, it was shown that the mean of fundamental frequency undergoes no increasing or decreasing trend during the first 53 days of life [56]. Besides this, the supralaryngeal VC is reconfigured towards a human vocal tract after the 3 months of age [55]. Therefore, newborns over 53 days old were not included in the current study.

### 2.2. Pre-Processing

Corwin et al. [40] described the four types of acoustic units that constitute a cry signal as expiratory phonation, expiratory hyperphonation, expiratory dysphonation, and inspiratory phonation. During the phonation, the vibrations of the newborn’s vocal folds generate sound, which is also referred to as voicing. The inspiratory cries are the “gasping” inhalation after the onset of crying that has enough power to cause vibrations in the vocal folds. Since the INSV episodes of the cry represent the laryngeal straitening of the ingressive air current, these cries have the potential to be a biomarker for diagnosis purposes [57]. The power needed for driving the expiratory phase of a cry is stored during the inspiratory phase. Expiration can be interpreted as a moderate decrement in the volume of the lungs [23]. Usually cries occur during this respiratory phase, so this segment is considered to contain the main information, while the inspiratory cries remain the less explored and cognizant type of the cry event by researchers. Although it has been reported that the restraint of the upper airway may lead to sudden infant death syndrome and apnea, and the inspiratory cry is believed to contain information leading to pain and distress [23], this type of cry has been often neglected in the study of NCDS [58]. Concisely, analysis of both expiratory and inspiratory cries is indispensable regarding the design of a comprehensive NCDS, and in this study, both expiratory and inspiratory phonation were included.

**Figure 1:**
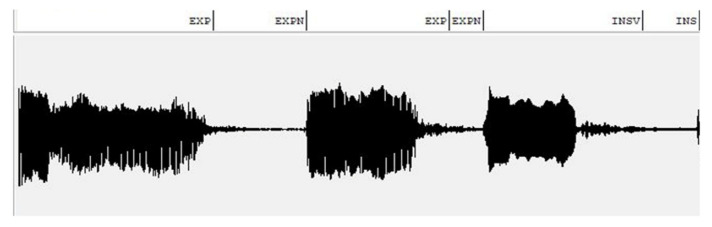
An example of a labeled cry signal via WaveSurfer. The X axis represents time, and the Y axis represents amplitude (please see Table A1 for description of the labels).

The cry samples in our dataset were labeled by a group of researchers. An example of the assigned cry signal units is depicted in Figure 1. Different segments of the cry signal have been margined, and matching labels have been attached via WaveSurfer (version 1.8.8, Stockholm, Sweden), presented in our previous works [10,54].

Table 2 represents the number of samples in each dataset as well as the number of samples separated for the test and training. In total, 68 newborns with one of the mentioned pathologies were included in the unhealthy subsection of the data, and 300 healthy newborns participated in this study. Each of these participants yielded a different number of samples in the dataset. An equal number of samples from the healthy group were selected to ensure a balanced analysis.

### 2.3. Feature Extraction

**Figure 2:**
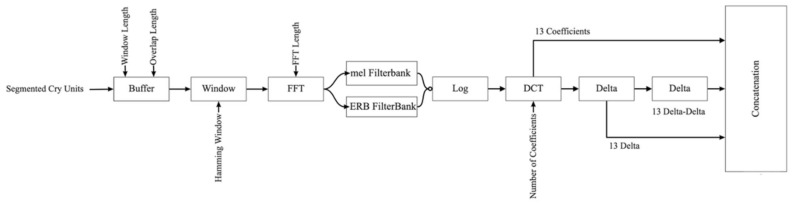
Framework of the proposed NCDS.

The extraction of appropriate acoustic features capable of pertinent signal representation plays a vital role in any audio classification problem. As discussed above, for the effectuation of a cry, glottal impulses proceed through the filtering carried out by the vocal tract [59]. With the aim of distinguishing between the source and filter of a cry, cepstral analysis employed, which enables a homomorphic transformation [60]. MFCCs were derived from the Mel Filter Banks, whereas GFCCs were obtained from the Gammatone Filter Banks, which are a representation of inner and external middle ear physiological transitions [61]. In other words, although the two approaches are based on from the human sound perception model, the GFCCs are coordinated to comprehend the physical alterations more effectively than the MFCCs, and better delineate the auditory system [62]. Both Gammatone and Mel-frequency representations of the cry signal were mapped into the cepstrum space for the feature extraction step. Figure 2 illustrates our framework; the proposed steps for the acquisition of each of these features are described in the following sections.

#### 2.3.1. Mel-Frequency Cepstral Coefficients

The calculation of MFCCs follows several steps. First, the preprocessed cry signal is pre-emphasized and divided into frames of 10 ms with a 30% overlap using the hamming window. The Fast Fourier Transform (FFT) then converts these frames to obtain the signal’s spectrum. In the next step, the spectrum is transformed to the Mel-frequency scale, which is a representation of perceived pitch. For this purpose, a filter bank consisting of 13 triangular Mel-spaced filters is employed. As a consequence of the vocal tract’s uniformity, the adjoining bands in the filter bank are inclined towards having correlated energy levels. Hence, a Discrete Cosine Transform (DCT) is imposed to decorrelate them and yield Mel-frequency Cepstral Coefficients. The Mel-scale of the frequency 𝑓 can be approximated as Equation (1).

𝑀(𝑓) = 1125 *ln*(1 + 𝑓/700) (1)

It was shown that the first thirteen coefficients could efficiently track the variations in the shape of the vocal tract during the generation of a sound by humans [63]. A similar procedure as in the previous works [10,64] was followed and the average statistical measure was used. The MFCCs hold information from one individual frame, and are therefore described as static features. In order to attain information on the fluctuations of the cry signal across multiple frames, the first and second derivatives of MFCCs are computed. Equation (2) gives the first derivative of the MFCCs for T consecutive frames (set equal to 2 in this study):

(2) $$\Delta c_{m}\left(n\right)=\frac{\sum_{i=-T}^{T}k_{i}c_{m}\left(n+i\right)}{\sum_{i=-T}^{T}\left|i\right|}\;\;\;\;\;\;\;\;\;\;\;\;\;\;\;\;\;\;\;\;\;\;$$

Here, the *m*th feature for the *n*th frame is represented by *c_m_*(*n*), and k_i_ denotes the *i*th weight. Calculating the first-order derivative of the delta coefficients yields the delta-delta coefficients. The total number of features in the MFCC feature set equals 39, including 13 MFCCs, 13 deltas, and 13 delta-deltas [65].

#### 2.3.2. Gammatone Frequency Cepstral Coefficients

Gammatone Frequency Cepstral Coefficients are a variant of MFCCs based on the biological response of the human auditory system. These features are extracted from the Gammatone filters with equivalent rectangular bandwidth (ERB) bands. Valero et al. [66] reported that the GFCC successfully performed non-speech audio classification tasks. It was also reported that the computation of the GFCCs was cost-efficient and has greater noise robustness compared to the MFCCs [10]. The procedure for obtaining the GFCCs is similar to the MFCC. The non-stationary cry signal was windowed into frames of 10 ms with 30% overlap. The hamming window was applied for this purpose. The Gammatone filter banks were then applied to the FFT of the cry signals, which was done in order to amplify the perceptually meaningful sound signal frequencies. Next, the output of the last step was mapped into the logarithmic space. Finally, the Discrete Cosine Transform (DCT) was applied to decorrelate the filters’ outputs and better mimic human loudness perception. The *m* coefficients from *N* Gammatone filters were then calculated via Equation (3).

(3) $$GFCC_{m}=\sqrt{\frac{2}{N}}\sum_{n=1}^{N}log\left(X_{n}\right)\;cos\left[\frac{\pi n}{N}\left(m-\frac{1}{2}\right)\right]\;\;\;\;\;\;\;\;\;1\leq m\leq M$$

where $X_{n}$ represents the corresponding energy of the *n*th band. Finally, the GFCC delta and delta-delta coefficients were derived, and the feature set comprised 39 coefficients matching the MFCC feature vector [66].

### 2.4. Features Fusion

By means of feature fusion, multiple feature sets are consolidated to create a single feature vector more robust than the individual feature vectors. Feature fusion can be undertaken in four different stages of the NCDS: (1) the data/sensor level; (2) the feature level; (3) the matching score level, and (4) the decision level [67].

In feature-level fusion, appropriate feature normalization, transformation, and reduction are employed in order to merge the features extracted from different sources into one feature set. The main benefit of feature-level fusion is the detection of correlated feature values generated by multiple algorithms, making it possible to introduce a new compressed set of salient features that can enhance classification accuracy. Therefore, CCA fusion at the feature level was utilized as the feature fusion strategy in this study [68].

#### Canonical Convolution Analysis (CCA)

Canonical convolution analysis handles the mutual statistical association between two feature sets by constructing a correlation criterion function. Subsequently, the canonical correlation regarding the criterion chosen in the last step was exploited, and discriminant vectors were forged so that the surplus information could be suppressed [69].

Suppose we take two feature sets X and Y of p×n  and q×n dimensions, respectively. In other words, for each *n*th sample of the dataset, p+q  features were extracted. In order to obtain information about all the relations across the feature sets, the overall covariance matrix, S, can be written as Equation (4):

(4) $$S=\left(\begin{array}{cc} cov\left(x\right) & cov\left(x,\;y\right)\\ cov\left(y,\;x\right) & cov\left(y\right) \end{array}\right)=\left(\begin{array}{cc} S_{xx} & S_{xy}\\ S_{yx} & S_{yy} \end{array}\right)$$

Apprehending the associations among the two sets of features may become challenging when they do not follow a steady pattern. CCA solves this challenge by finding the linear combinations, $X^{⋇}=W_{x}^{T}X$ and $Y^{⋇}=W_{y}^{T}Y$, and attaining the maximum pair-wise correlations, which is facilitated via Lagrange multipliers. The pair-wise correlation is defined as Equation (5).

(5) $$corr\;\left(X^{⋇},\;Y^{⋇}\right)=\frac{cov\left(\;X^{⋇},\;Y^{⋇}\right)}{var\left(X^{⋇}\right)\;\;.\;\;var\;\left(Y^{⋇}\right)}$$

Finally, feature-level fusion is achieved by the concatenation of the transformed feature sets as in Equation (6):

(6) $$Z=\left(\begin{array}{c} X^{⋇}\\ Y^{⋇} \end{array}\right)=\;\left(\begin{array}{c} W_{x}^{T}X\\ W_{y}^{T}Y \end{array}\right)={\left(\begin{array}{cc} W_{x} & 0\\ 0 & W_{y} \end{array}\right)}^{T}\left(\begin{array}{c} X\\ Y \end{array}\right)$$

*Z* represents the Canonical Correlation Discriminant Features (CCDFs) [70]. The GFCC and MFCC feature sets’ fusion results, constituting 39 features each, yielded a feature vector with 60 features representing the cry signals. In this study, the performance of the fused features was compared to the individual feature sets, as well as their concatenation.

### 2.5. Classification

Classification assigns class labels to the given data points. The evaluation of the extracted feature sets was performed by classification. Two different classifiers were implemented in the current study. The first classification method was SVM, which is conventional in NCDS research. Moreover, the LSTM neural network was also employed. These classifiers and the HPO methods associated with each one are introduced in the following sections.

#### 2.5.1. Support Vector Machine (SVM)

SVMs are prevalent in the analysis of audio signals. SVM is known as a supervised ML classification method that draws a hyperplane to maximize the marginal distance between the classes of data. The support vectors represent boundary feature points and form the basis for classification. A kernel function handles the nonlinearity of the data [71]. A Gaussian Kernel was implemented, which assumes that similar feature points were located in close vicinity and considers the Euclidean distance between x and x_i_. In this study, the box constraint and kernel scale were tuned as the HPs of the SVM model.

#### 2.5.2. Hyperparameter Optimization (HPO)

In any classification problem, the goal is to achieve high performance while keeping the errors to a minimum; therefore, HPO methods have been introduced. The several approaches to the HPO of an ML classifier include grid search, random search, and BHPO, which have omitted the need for human intervention to tune the classifier’s HPs. The significance of the HPO is that each configuration is designed to fit its corresponding task. The main function of any given HPO method is to attain an optimum value for each HP from a set of finite values that minimizes the loss or maximizes the objective function. However, there are always downsides to each method, such as the high computational costs associated with the NN HPO and the probability of facing the curse of dimensionality [72].

The acquisition function and probabilistic surrogate model are the basis of the BHPO. The acquisition function enables a BHPO model to be updated in correspondence to it iteratively, defined as Equation (7) [73]:

(7) $$x^{*}=\underset{x\in X}{argmin}f\left(x\right)$$

In every iteration, the model is updated based on new HPs and the corresponding model performance. Once the predefined number of iterations is reached, the best observed HPs are announced, as are the optimal observed values for the objective function. As will be seen in the following sections, BHPO often achieves better results than the other two HPO methods introduced.

#### 2.5.3. Long Short-Term Memory (LSTM)

**Figure 3:**
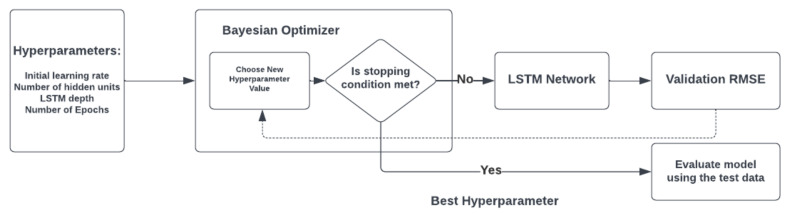
General Bayesian hyperparameter optimization process.

Recurrent Neural Networks (RNNs) have been shown to be propitious in the analysis of both single data points and sequential data, such as acoustic inputs. A feedback loop connects the input of RNN to its output, allowing them to model the dependencies in time series. Long Short-Term Memory (LSTM) networks are a type of RNNs with memory cells capable of learning, keeping, and forgetting data. By means of this memory cell, LSTMs function well with both short-term and long-term features [74]. Since the generation of the cry signal is intrinsically dynamic, RNNs may prove functional in their acoustic modeling. However, the challenge arises from the complexity of the training and tuning of hyperparameters of these networks. In order to overcome this challenge, the HPO methods were implemented to find and choose the optimal HPs. As mentioned above, Bayesian optimization requires fewer iterations than the grid search method to achieve the optimal values for the HPs in neural networks. The general task of HP acquisition by the BHPO is depicted in Figure 3.

The range of each hyperparameter was pre-determined in order to exploit the full potential of the HPO methods, as is shown in Table 3.

The activation function for this LSTM configuration is the hyperbolic tangent function (tanh). The hyperparameters included in this set of experiments were initial learning rate, number of hidden units, maximum epochs, and the depth of the LSTM architecture. Increasing the depth of the network was accompanied by higher computational costs, and all the best results were achieved when using only one layer. The loss and root mean squared errors were calculated for each run of the optimization process. The optimization was performed in 30 evaluations for each set of features, and the parameters that maximized the overall accuracy were chosen for each configuration.

## 3. Evaluation

This study aimed to differentiate pathologic infants from healthy infants and employed the GFCC and MFCC features with the LSTM and SVM classifiers. A wide range of pathologies were included in these experiments in order to achieve a comprehensive NCDS, which is able to act as an early alert given the lack of medical experts and access to expensive and extensive laboratory experiments. Different experiments were conducted with the proposed feature vectors, their combination, and their CCA fusion. Following the feature extraction step is classification. There are two approaches to validating the classifier’s performance after training: holdout and cross-validation. The data were split into 70% training and 30% unseen testing data for both classifiers. For the SVM classifier, a 5-fold cross-validation was conducted on the training data, whereas for the LSTM classification, a holdout validation approach was chosen with 20% of the training data with a frequency of once every 10 iterations, because of the different natures of the classifiers. For the k-fold cross-validation, the data were split into *k* partitions, *k*−1 folds of which were used for training and one fold for testing in each iteration. This procedure was repeated up to the point at which each of the *k* folds was marked as the test fold. Finally, the results of grid search and BHPO for each architecture were compared.

The discriminatory performance of an NCDS in a binary problem can be represented by a contingency matrix, as shown in Table 4. The task of the NCDS in our paper was to detect the pathological neonates amid the healthy. In order to appraise how well the system performed its role, the evaluation measures were introduced and computed. Practically, the most convenient evaluation measure is the accuracy, which is equivalent to the proportion of correctly predicted samples over all the observations. The accuracy measure benefits from both calculation and apprehension simplicity; however, the lack of informativeness as well as the fewer concessions towards the minority calls for the implementation of more evaluation measures [75].

One solution is to evaluate the NCDS performance without considering the true negative case, which will introduce a measure named precision. Precision, or Positive Predictive Value (PPV), is the ratio of true pathologic cases among the samples predicted as healthy. Another measure is recall, or sensitivity, which refers to the probability of recognizing a truly pathologic case by NCDS. The F-score and Matthews’ Correlation Coefficient (MCC) were reported to be more instructive in binary classification problems. F-score is a function of both recall and precision, and indicates the inclusive performance of the system and is equal to the harmonic mean of precision and recall [76]. The specificity measure denotes the true negative rate, and it indicates the true healthy samples correctly identified by the NCDS [77].

The MCC is a highly informative evaluation measure when used in problems such as NCDS designs, since it accounts for all the information in a contingency matrix. The MCC, Equation (8), gives a value in the range of [−1, +1], where the misclassified performance results in negative values, and the higher values in the positive range signify better performance in terms of classification [78,79]. In this study, a high acceptance value of +0.50 was set to evaluate the classification.

(8) $$MCC=\frac{TP\times TN-FP\times FN}{\sqrt{\left(TP+FN\right)\left(TN+FP\right)\left(TP+FP\right)\left(TN+FN\right)}}$$

## 4. Results

This section presents the results of evaluating different architectures with multiple measures in Table 5, Table 6, Table 7, Table 8, Table 9, Table 10, Table 11 and Table 12. Regarding the evaluation measures introduced, higher values for each measure translate into the better performance of the system. In this study, four sets of experiments were conducted: 1. Evaluate the NCDS performance with default/random search hyperparameter configuration of the classifiers. 2. Evaluate the NCDS performance with grid search HPO. 3. Evaluate the NCDS performance with BHPO. 4. Compare the performance of the system with different iterations of HPO for each method, ranging from 30 iterations to 100 iterations for SVM and different numbers of neurons for the LSTM.

Each of the feature vectors were evaluated with the SVM classifiers, which are shown in Table 5, Table 6, Table 7 and Table 8. In this step, the evaluation of system performance was undertaken by three different settings of the classifier: 1. Default settings. 2. Grid search optimization. 3. BHPO. The same procedure was repeated for the LSTM classifier, and 30 iterations of each HPO method were performed.

First, the results related to using the SVM classifier as a baseline to compare the results of the next steps are discussed. Table 5 represents the results for the MFCC feature set for the INSV and EXP datasets. The use of HPO similarly increased the evaluation measures across both datasets. Moreover, BHPO achieved a very similar or better performance except for in the recall measure. The highest accuracy and F-score for the EXP dataset were 87.37% and 86.64%, respectively; both were obtained through BHPO. This experiment yielded a better performance with the INSV dataset, and yielded 89.05% for the accuracy measure, which was again achieved through BHPO. However, grid search had a slight superiority in terms of the F-score, and achieved 89.24%.

Table 6 presents the results of evaluating the GFCC feature set with the SVM classifier. By briefly looking at Table 5 and Table 6, it can be seen that the MFCC feature set outperformed the GFCC feature set across both datasets. Similar to all the other feature sets, the best results in terms of F-score and accuracy in relation to the EXP dataset for the GFCC feature set were achieved through BHPO. In a general sense, the combination of the GFCC with the SVM yielded better results with the INSV dataset compared to the EXP dataset. The GFCC features’ highest accuracy and F-score were 85.51% and 85.88%, respectively; both were achieved with BHPO and INSV dataset.

In the next step, the GFCC and MFCC feature sets were combined to evaluate the NCDS performance under these conditions. As for the EXP dataset, the concatenated feature set could increase the accuracy and F-score measures by 1% and 1.7%, respectively, compared to the best results of the last two feature sets. The highest results for the EXP dataset were 88.41% and 88.30% for accuracy and F-score, respectively. The performance of the NCDS with this configuration for the INSV dataset was very similar to that for the MFCC feature set used individually, and there was a slight improvement in the evaluation measures (Table 7).

As a final experiment with the SVM classifier, the GFCC and MFCC feature sets—each containing 39 elements—were fused, and the feature vector was reduced to 60 elements, which was a more than 25% reduction in the size of the feature space. Since the size of the feature space was reduced, it might be expected that we see a rather small drop or a similar performance across the evaluation measures with this experiment compared to with the EXP dataset. However, as can be seen from Table 8, not only were the overall best results in terms of accuracy and F-score maintained, but they were also increased by about 1%. The results for the INSV dataset show the new highest accuracy and F-score across all the experiments with the SVM classifier, with 89.96% and 90.27%, respectively. For the EXP dataset, compared to the best results in terms of accuracy and F-score in previous experiments, the fusion of the features decreased the performance of the NCDS by 0.7% and 0.35%, respectively.

After evaluating different aspects of the NCDS with the SVM classifier, the study proceeded to design an LSTM configuration to differentiate pathologic newborns from the healthy group. The same procedure of the experiments as with the SVM classifier was followed, and the system was evaluated with each feature configuration separately. The performances of all feature sets were improved considerably by using the LSTM classification method. The MFCC feature set achieved the highest accuracy and F-score of 99.03% and 99.05%, respectively, with the LSTM classifier for the INSV dataset, which is a nearly 10% improvement compared to the SVM method. As can be seen from Table 9 and Table 10, the performance of the GFCC feature set was slightly better than the MFCC feature set with the LSTM classifier for the EXP dataset, and vice versa for the INSV dataset. Both HPO methods worked marvellously with the LSTM classifier; however, they were not efficient in terms of run-time, which will be compared in the Discussion section.

The best accuracy and F-score achieved by the GFCC feature set were 99.45% and 99.44%, respectively, whereas the MFCC obtained 99.33% for both measures. The mentioned results were accomplished for the EXP dataset. It is noteworthy to mention that both feature sets attained 100.00% for specificity and precision measures. Moreover, the MCC measure has acquired a high value for the BHPO with EXP dataset for both feature sets, which indicates close to perfect classification quality (Table 10).

Even though the state-of-the-art performance of both individual feature sets through HPO methods leaves little room for improvement, it is still beneficial to study the behavior of the system by the combination of the two feature vectors to assess their efficacy compared to the SVM classifier. As can be deduced from Table 11, the performance of the NCDS was degraded by simply concatenating the feature sets, which may translate to lower uniformity of the feature space. The highest accuracy and F-score achieved with this experiment belonged to the INSV dataset, which reached 98.99% and 99.00%, respectively.

Table 12 constitutes the results of the next experiment with the LSTM classifier. The system’s performance was better than the concatenation framework since the CCA fusion removes the redundant features and helps improve the uniformity of feature space. This experiment showed the best performance in assessing the INSV dataset among all the previous experiments for all of the evaluation measures, specifically reaching 99.86% for both F-score and accuracy and 1.00 for the MCC measure. As for the EXP dataset, the GFCC feature set outperformed both combinational feature sets in terms of all evaluation measures.

**Figure 4:**
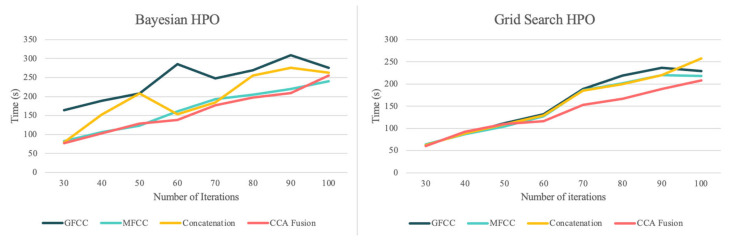
Elapsed time (seconds) regarding the different iterations of hyperparameter optimization methods for the SVM classifier.

In the previous section, the evaluation results regarding each feature set and classifier combination were extensively discussed; now, the discussion is undertaken from the perspective of the computational cost. For this matter, the run-time was selected as an indicator. It should be noted that in the case of the joint feature sets, namely, concatenation and CCA fusion, the given run-times include the process of concatenation and fusion, and not only the time corresponding to the HPO process. The elapsed times for the extraction of the GFCC and MFCC feature sets were 558.31 and 836.16 s, respectively, which suggests the GFCC feature set requires lower computational costs; other researchers have also mentioned the same results [66]. Figure 4 compares the run-times of the grid search HPO and BHPO methods for different iterations of each one when applied to the SVM classifier.

**Figure 5:**
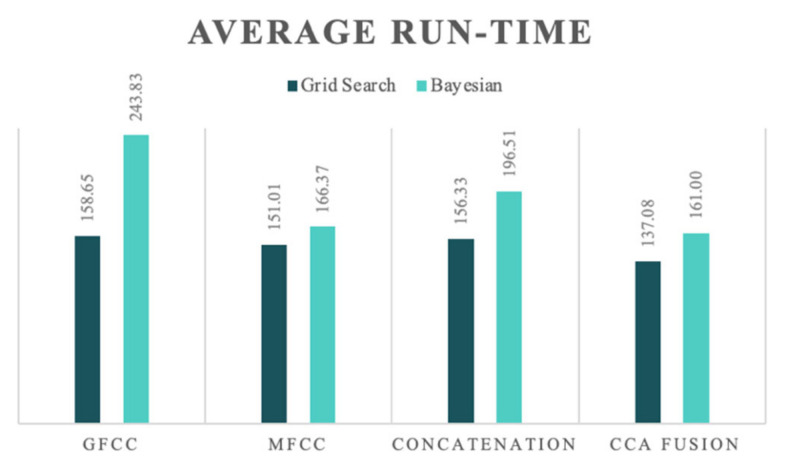
Average run-times for evaluating different iterations of hyperparameter optimization for the SVM classifier.

The comparison between run-times regarding each feature set firstly confirms that the CCA fusion method results in a more homogenous feature space, and reduces run-times until they are lower than the run-time for the individual feature sets, which is consistent in both HPO methods. As can be seen, BHPO resulted in the higher performance of the system and required longer run-times. In order to better illustrate this comparison, Figure 5 presents the average run-times of the two HPO methods for each NCDS configuration for a more detailed evaluation.

**Figure 6:**
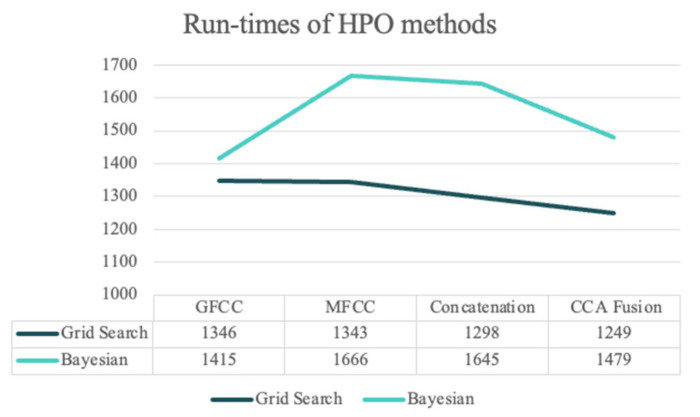
Comparing run-times for the two HPO methods of LSTM for different experiments.

Figure 6 shows the elapsed times (in seconds) for the grid search and BHPO methods for the LSTM classifier. Since the process of HPO for the NNs is highly time-consuming compared to the machine learning models, only 30 iterations of HPO were performed for this experiment. The results show that CCA fusion requires the shortest run-time out of all other feature sets for the grid search HPO, similar to the SVM HPO methods; the run-times regarding the grid search method were lower than for BHPO. It should be noted that the number of trials for both methods was limited to 30; BHPO can achieve satisfactory results with this number of iterations, whereas grid search often requires a much greater number of trials. In summary, the proposed NCDS in this study accomplished desirable results across all the experiments in terms of performance and computational costs, and the longest elapsed time was less than 1700 s simultaneously.

## 5. Discussion

The design of the NCDS is a challenging problem for every researcher aiming to study newborn cry characteristics, regardless of the purpose the NCDS aims to serve. This challenge is even more significant regarding the sensitive subject of detecting pathologic newborns. The NCDS designs are not developed enough compared to the other acoustic scene recognition systems or speech analysis applications; there is still a need for further studies in this field, which is mainly due to the fact that datasets are very limited in terms of the number of samples. This is due to certain limitations, such as the fact that the chances of having a newborn diagnosed with a specific pathology in any given duration of conducting a clinical study are not predictable. Therefore, there may not be sufficient samples from each given pathology group; ensuring the ethical and technical standards required to collect and use the cry samples in a database calls for extreme measures. In this regard, by segmenting each cry recording into multiple expiratory and inspiratory episodes, two datasets of EXP and INSV were formed. As mentioned above, the areas of the world that suffer the most from infant mortality are less developed and lack a sufficient number of expert physicians. Thus, it is vital to keep the design as simple as possible so that expensive hardware would not be required to achieve high performance.

One other aspect of the proposed study is that by employing MFCC and GFCC features, the cry signal is investigated both from the speech processing and non-speech audio processing perspectives. As was previously discussed, MFCCs have proved to be powerful discriminators, especially in speech processing tasks, while GFCCs have shown even better performance and robustness in non-speech audio applications. For the first time in newborn cry analysis a CCA fusion at the feature level was performed in order to make the feature space homogenized and omit redundant information. By looking at the results of run-times in the previous section, it can be seen that CCA fusion homogenized the feature space in a way that the fused feature vectors required less time for optimization, even when compared to the single feature vector of GFCC. This shows that although the fused vector had 60 elements, it was still optimized faster than a 39-element feature vector, even with the time required for fusion included. This is rather an interesting finding that shows potential for many further applications with the inclusion of features from various modalities in this field. Not only were the HPO run-times reduced, but also, the results were improved by reducing the number of features by about one fourth.

Since the challenge is to detect a pathologic newborn and alert the newborn caregivers and medical experts, it is worth tolerating higher run-times in order to obtain a more accurate diagnosis and benefit from the HPO methods. The other important factor here is that the NCDS cannot afford to misdiagnose a pathologic newborn as a healthy one, so the focus should be on achieving a high hit rate (recall) and F-score measure, which are the indicators of a low miss rate. This study proposed two different designs with respect to the runtime and performance trade-off. Firstly, using the SVM classification method, a simplistic design was proposed, which requires minimal run-time and could work with commercial hardware. It was shown that by implementing the HPO methods, a similar performance to the complex state-of-the-art designs with up to 90% F-score for the SVM could be achieved. Moreover, our LSTM design, which only has a one-layer depth, was able to achieve better F-scores than similar or more complex works in the literature using the proper HPO, with improvements of 99.86% and 99.45% for INSV and EXP datasets, respectively. This study also offers an extensive evaluation of the HPO factors and methods in addition to the primary goal, achieving high diagnostic power. Additionally, the powerful discriminatory role of inspiratory cries, which are neglected in most NCDS studies, is highlighted here, as is the success of our design with the EXP dataset, which worked even better with this dataset.

**Figure 7:**
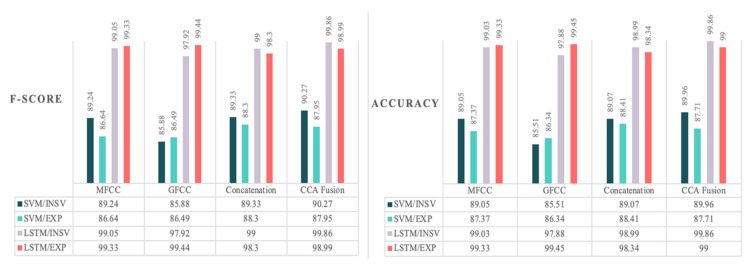
Summary of the best results achieved with the conducted experiments in terms of accuracy and F-score measures.

Finally, the high number of pathology groups included in this study makes it a comprehensive framework capable of a more reliable diagnosis, since the medical staff could suggest that the newborn does not suffer from the given list of pathologies. Figure 7 gives a visual summary of the best results achieved by each experiment in terms of F-score and accuracy. These results imply the similar performances of the NCDS in terms of both F-score and accuracy measures, which indicates that discussing the F-score measure alone would be sufficient.

In order to evaluate the results from another perspective and further explore the potential of both HPO methods and CCA fusion, another experiment was designed wherein the performance of the NCDS could be investigated with different HPO iterations of 30 to 100 (rising by steps of 10) on the EXP dataset. The average of all evaluations (eight experiments) across all measures is reported in Table 13. As can be inferred from the results, both HPO methods enhanced the system performance in terms of accuracy, recall, F-score and MCC measures. Several patterns were observed when conducting these experiments. Firstly, the performance of the BHPO method was superior to that of the grid search method across all the measures, except for the recall measure. Even though the recall measure represented an exception to the mentioned pattern, the highest recall was achieved through the BHPO method with the fusion of features, which was 90.25%. Secondly, the best performance in terms of accuracy, MCC, and F-score was achieved using the CCA fusion framework. Finally, it can be deduced that although CCA fusion slenderized feature space, the performance of the NCDS was not considerably aggravated, and was even increased in terms of the F-score measure.

As was previously discussed in the Results section, the evaluation measures showed exceptional performance with the LSTM classifier. Therefore, to better demonstrate the power of LSTM in the NCDS design and validate the surprisingly high performance of the system, a final experiment was mapped out. In this experiment, the LSTM classifier was manually tuned for only one HP: the number of hidden neurons. For each feature set, the number of hidden neurons was changed from 2 neurons to half the size of each feature vector, e.g., 30 neurons for the fusion feature set with 60 elements for each sample. Table 14 presents the average of each evaluation measure used in the successful attempts with manual search methods for each feature set. Therefore, if the only parameter being tuned is the number of hidden neurons, the system’s performance undergoes a bearable decline in exchange for lowering the computational costs. Moreover, as can be seen from the results, the best evaluation measures belonged to the CCA-fused feature set (except for the recall measure), which are 96.62% and 96.58% for accuracy and F-score, respectively. Therefore, by manually tuning only one HP, the system was capable of achieving up to a 96.58% average F-score, which translates to the high classification power of the LSTM classifier compared to the SVM, and the potential for an even better performance if other HPs are tuned as well.

So far, the experiments in this study have been discussed and compared in terms of performance, classification power, and run-times. There are various tools and frameworks for the study of audio signals, which have resulted in many different applications and publications. Among these frameworks, many different machine learning and deep learning methods have been explored. It is worthwhile to compare the performance of the proposed NCDS with other similar works or architectures that analyze either newborn cry signals or other audio signals. In a recent study [80], environment sounds were classified through different models including SVM, LightGBM, XGBoost (XGB) nd CatBoost classification frameworks, employing time and frequency domain features and their combination. They were able to get 87.3% as their highest accuracy measure when using the LightGBM framework, and through the alteration of the gain factor, whereas their baseline classifiers yielded 66.7% for KNN, 67.5% for SVM, 72.7% for baseline Random Forest (RF), and 81.5% for their joint feature set with RF classifier. In another study [81], speech signals were employed to diagnose Parkinson’s with the use of RF, Decision Tree (DT), KNN, XGB, and Naïve Bayes (NBC) classifiers. The results show that the classifiers’ performances ranked as follows: XGB achieved 96.61% for the accuracy measure, and KNN, RF, DT and NBC achieved accuracies of 94.91%, 88.13%, 86.44%, and 67.79%, respectively. The study of Singhal et al. [82] classified music genres with Logistic Regression (LR), KNN, SVM, XGB, and RF classifiers. They also explored the effect of HPO on the RF classifier only, where the results were enhanced about 13% for accuracy and reached 98.8%. However, they did not discuss the HPO methods and trends. The highest result was achieved when using both RF and XGB classifiers—99.6% for both frameworks. The study of Kim et al. [83] explored a very similar framework to the one presented in this study for the analysis of beehive sounds through MFCCs, a Mel spectrogram and constant-Q transform features, with RF, XGB, CNN, and SVM classifiers. The highest accuracy was achieved through the combination of MFCC features with the XGB classifier, reaching 87.36%. Their VGG-13 classification showed very promising results, with 96% for the F-score measure. Lahmiri et al. [47] designed an NCDS for the purpose of detecting pathologic newborns with cepstrum features and multiple NN classifiers. By implementing LSTM classification, they were able to achieve an accuracy of 83.89% and 80.18% for the EXP and INSV datasets, respectively. Another work worth mentioning in this field is that by Matikolaei et al. [84], wherein the proposed NCDS served the same purpose as in our study. The authors combined the MFCC with the auditory-inspired amplitude modulation features, and fed them into an SVM classifier; they attained 80.50% for the accuracy measure. Kumaran et al. [45] focused on the recognition of emotions; they combined the GFCC with the MFCC feature sets and employed C-RNN classification. They used a different architecture of LSTM than in our study, with the addition of a convolutional network, and the highest F-score yielded by their design was 79%. In another emotion recognition study, the MFCC features were employed with a combined CNN-LSTM architecture, and the highest accuracy of 87.4% was reported with the use of HPO methods, wherein they tuned learning rate and batch size [46]. Given the results of the mentioned studies, our NCDS designs proved to be successful and introduced novelty to the study of newborn cries with the purpose of detecting pathologic infants. Our study proposed a simplistic design using the SVM classifier that benefits from BHPO; we showed it could achieve results similar to (and even better than) the state-of-the-art of NCDS employing NNs, which in the literature reached 90.27% for the F-score measure. Our second framework of LSTM classification with BHPO obtained up to 99.86% for the F-score measure, which is remarkable in the study of pathologic newborn cry signals. However, our system was outperformed by a design that implements DFFNN, since it was able to achieve 100% for both datasets of EXP and INSV [85].

In the design of the LSTM, the main concern was to prevent the model from becoming complex, and it employed only one hidden layer with a low number of hidden units. Both these achievements owe their success to the CCA fusion of the GFCC and MFCC feature sets, which not only enhances the overall performance, but also lowers the run-time by homogenizing the feature space and marks out the optimal feature set.

In summary, the presented results of this study suggest that a fusion of MFCC and GFCC features fed to deep and machine learning classifiers attains a higher performance compared to previous studies on detecting pathologic newborns. This framework is proposed as a non-invasive tool for aiding the expeditious detection of pathologic infants. There is still a vast ocean of unexplored ideas and architectures to be implemented in the study of pathological newborn cry signals, which is beyond the scope of this study. In future works, exploring more deep learning and machine learning designs such as DCNNs, and further exploring fusion techniques, especially at the decision level, such as the matching score method, would be of interest. Furthermore, studying more acoustic features and combining them with different classifiers would be worthwhile in order to highlight the efficacy of existing research on pathologic newborn cry signals.

## 6. Conclusions

The cry of infants has been recognized as a biomarker in the detection of pathologies for the purpose of early diagnosis. The presented study aimed to propose a comprehensive NCDS that distinguishes between healthy and pathologic cries regardless of the reason for crying, race, and gender. Our proposed system outlines the feature of the GFCC and its delta coefficients, which efficiently capture the dynamic nature of the cry signal and its periodic pattern. Moreover, the feature set used in this study includes the MFCC, which is well-known for its strong performance in many acoustic applications. These features were fed individually and fused into the LSTM and SVM classifiers, which belong to two different families of classifiers. In the next step, an extensive study of HPO methods for grid searching and BHPO for both classifiers was performed in order to improve the performance of NCDS. The LSTM was able to achieve a very high performance metric of 99.86% when applied to inspiratory cry signals in terms of both accuracy and F-score, owing to its capability to learn from sequential data. Furthermore, LSTM outperformed the optimized SVM when applied to both the studied datasets. All of the results obtained by the two proposed classifiers show potential for use in the investigation of pathologic infant cries.

This study contributed to the development of NCDS with the aim of designing a first alert for medical experts; it showed that healthy and pathologic infants have different cry patterns, which can be used as biomarkers. Regarding the results of this study, the proposed framework can be used as a non-invasive diagnostic tool without the need for high-end hardware and technologies.

## Figures and Tables

**Table 1:** Description of dataset and participants.

Demographic Factors	Specification
Gender	Female and Male
Babies’ Ages	1 to 53 days old
Weight	0.98 to 5.2 Kg
Origin	Canada, Haiti, Portugal, Syria, Lebanon, Algeria, Palestine, Bangladesh, Turkey.
Race	Caucasian, Arabic, Asian, Latino, African, Native Hawaiian, Quebec.
Cry Stimulus	Discomfort, lack of sleep, wet diaper, pain, fear, colic, reflux, birth cry, hunger.
Healthy/Pathology Group	Healthy, dyspnea, fever, gastroschisis, grunting, hyperbilirubinemia, hypoglycemia, hypothermia, intrauterine growth retardation, jaundice, kidney failure, meconium aspiration syndrome, meningitis, myelomeningocele, respiratory distress syndrome, retraction, seizure, sepsis, tachypnea, thrombosis in vena cava, vomit.

**Table 2:** Number of samples in each dataset for training and test.

	No. of Healthy	No. of Pathologic	No. of Train Samples	No. of Test Samples
EXP	3005	3005	4207	1803
INSV	3620	3620	5068	2172

**Table 3:** Predefined ranges for the hyperparameter optimization of the LSTM.

Parameter	Selected Range
Initial learning rate	[0.001, 1] (logarithmic steps)
Number of hidden units	[2, 39]
Number of Epochs	[100, 500]
Depth	[1, 3]

**Table 4:** Contingency matrix for the evaluation of NCDS.

	True Class			
**Predicted class**		Pathologic	Healthy	Measures
	Pathologic	True Positive	False Positive	Precision $\frac{TP}{FP+TP}$
	Healthy	False Negative	True Negative	Negative Predictive Value $\frac{TN}{FN+TN}$
	Measures	Recall $\frac{TP}{TP+FN}$	Specificity $\frac{TN}{FP+TN}$	Accuracy $\frac{TP+TN}{TP+FP+FN+TN}$

**Table 5:** Results of evaluating the MFCC feature set classification with SVM.

	MFCC	Accuracy	Recall	Specificity	Precision	F-Score	MCC
**EXP**	Random Search	72.16	62.93	81.26	76.80	69.17	0.45
	Grid Search	86.34	88.09	84.63	84.97	86.49	0.73
	Bayesian	87.37	82.55	92.11	91.17	86.64	0.75
**INSV**	Default	79.38	78.36	80.45	80.73	79.53	0.59
	Grid Search	88.90	90.09	87.65	88.40	89.24	0.78
	Bayesian	89.05	88.72	89.40	89.74	89.23	0.78

**Table 6:** Results of evaluating the GFCC feature set classification with SVM.

	GFCC	Accuracy	Recall	Specificity	Precision	F-Score	MCC
**EXP**	Random Search	67.42	57.59	77.11	71.27	63.70	0.35
	Grid Search	83.75	86.06	81.48	82.08	84.02	0.74
	Bayesian	84.49	86.39	82.62	83.05	84.69	0.69
**INSV**	Random Search	76.57	73.53	79.74	79.14	76.23	0.53
	Grid Search	85.26	86.23	84.24	85.12	85.67	0.71
	Bayesian	85.51	86.25	84.73	85.52	85.88	0.71

**Table 7:** Results of evaluating the concatenation feature set classification with SVM.

	Concatenation	Accuracy	Recall	Specificity	Precision	F-Score	MCC
**EXP**	Random Search	75.49	68.58	82.29	79.25	73.53	0.51
	Grid Search	87.85	87.93	87.78	87.64	87.78	0.76
	Bayesian	88.41	88.13	88.68	88.47	88.30	0.77
**INSV**	Random Search	81.06	81.03	81.09	81.75	81.38	0.62
	Grid Search	88.59	88.40	88.79	89.19	88.79	0.77
	Bayesian	89.07	89.57	88.55	89.10	89.33	0.78

**Table 8:** Results of evaluating the CCA fusion feature set classification with SVM.

	CCA Fusion	Accuracy	Recall	Specificity	Precision	F-Score	MCC
**EXP**	Random Search	81.43	81.59	81.28	81.11	81.35	0.63
	Grid Search	85.31	87.20	83.46	83.86	85.49	0.71
	Bayesian	87.71	90.35	85.11	85.68	87.95	0.76
**INSV**	Random Search	88.09	88.43	87.74	88.29	88.36	0.76
	Grid Search	88.28	89.14	87.38	88.07	88.60	0.77
	Bayesian	89.96	91.14	88.74	89.43	90.27	0.80

**Table 9:** Results of evaluating the MFCC feature set classification with LSTM.

	MFCC	Accuracy	Recall	Specificity	Precision	F-Score	MCC
**EXP**	Random Search	76.59	95.98	57.49	69.00	80.28	0.58
	Grid Search	97.78	95.53	100.00	100.00	97.71	0.96
	Bayesian	99.33	98.66	100.00	100.00	99.33	0.99
**INSV**	Random Search	95.17	96.58	93.69	94.12	95.33	0.90
	Grid Search	96.09	92.34	100.00	100.00	96.02	0.92
	Bayesian	99.03	98.56	99.53	99.55	99.05	0.98

**Table 10:** Results of evaluating the GFCC feature set classification with SVM.

	GFCC	Accuracy	Recall	Specificity	Precision	F-Score	MCC
**EXP**	Random Search	84.53	75.20	93.72	92.19	82.83	0.70
	Grid Search	96.56	93.07	100.00	100.00	96.41	0.93
	Bayesian	99.45	98.88	100.00	100.00	99.44	0.99
**INSV**	Random Search	96.09	92.34	100.00	100.00	96.02	0.92
	Grid Search	97.88	97.66	98.12	98.19	97.92	0.96
	Bayesian	97.51	95.14	100.00	100.00	97.51	0.95

**Table 11:** Results of evaluating the concatenation feature set classification with SVM.

	Concatenation	Accuracy	Recall	Specificity	Precision	F-Score	MCC
**EXP**	Random Search	89.24	97.54	81.06	83.54	90.00	0.80
	Grid Search	96.23	92.40	100.00	100.00	96.05	0.93
	Bayesian	98.34	96.76	99.89	99.88	98.30	0.97
**INSV**	Random Search	98.48	97.21	99.81	99.81	98.49	0.97
	Grid Search	98.85	97.75	100.00	100.00	98.86	0.98
	Bayesian	98.99	98.11	99.91	99.91	99.00	0.98

**Table 12:** Results of evaluating the CCA fusion feature set classification with SVM.

	CCA Fusion	Accuracy	Recall	Specificity	Precision	F-Score	MCC
**EXP**	Random Search	96.73	93.74	99.67	99.64	96.60	0.94
	Grid Search	97.34	97.65	97.03	97.00	97.33	0.95
	Bayesian	99.00	98.55	99.45	99.44	98.99	0.98
**INSV**	Random Search	96.09	92.61	99.72	99.71	96.03	0.92
	Grid Search	98.16	97.57	98.78	98.81	98.19	0.96
	Bayesian	99.86	99.73	100.00	100.00	99.86	1.00

**Table 13:** Results for the average of the evaluation measure for different iterations of hyperparameter optimization ranging from 30 iterations to 100 iterations.

		Accuracy	Recall	Specificity	Precision	F-Score	MCC
**GFCC**	Bayesian	84.50	85.09	83.91	83.91	84.49	0.69
	Grid Search	83.75	86.06	81.48	82.08	84.02	0.68
**MFCC**	Bayesian	86.86	83.43	90.24	89.89	87.77	0.74
	Grid Search	86.34	88.09	84.63	84.97	86.49	0.73
**Concatenation**	Bayesian	88.18	87.90	88.46	88.25	88.07	0.76
	Grid Search	87.67	87.95	87.38	87.31	87.62	0.75
**Fusion**	Bayesian	87.92	90.25	85.63	86.10	88.12	0.76
	Grid Search	85.41	87.08	83.76	84.10	85.56	0.71

**Table 14:** The averages of evaluation measures for the manual tuning of the hidden neurons for the LSTM classifier with each feature.

	Accuracy	Recall	Specificity	Precision	F-Score	MCC
MFCC	89.02	94.23	83.89	89.16	90.54	0.80
GFCC	89.79	88.00	91.56	94.05	90.01	0.81
GFCC + MFCC	95.33	95.95	94.72	95.79	95.59	0.91
CCA Fusion	96.62	95.89	97.34	97.34	96.58	0.93

## Data Availability

Not applicable.

## Appendix A

**Table A1:** A comparative overview of selected NCDS designs for pathological newborn cry diagnostics.

Study	Pathologies	Evaluation Measures	HPO	Feature Fusion	Machine/Deep Learning Methods	Outcome
Matikolaie et al. [10,36,84]	RDS vs. healthy, septic vs. healthy, collection of pathologies vs. healthy.	Recall, precision, F-score, accuracy.	---	Concatenation	PNN, SVM, DT, Discriminant Analysis.	F-score Sepsis: 86%. Multi-Pathology: 80%. RDS: 68.40%
Kheddache et al. [28]	Hyperbilirubinemia, vena cava thrombosis, meningitis, peritonitis, asphyxia, lingual frenum, IUGR-microcephaly, tetralogy of fallot, gastroschisis, IUGR-asphyxia, RDS.	Accuracy.	---	Concatenation	PNN.	Accuracy: 88.71%.
Lahmiri et al. [47,85,86]	Central nervous system complications, chromosomal abnormalities, congenital cardiac anomalies, blood disorders.	Accuracy, recall, specificity.	---	Concatenation	CNN, LSTM, DFNN, SVM, NBC.	Accuracy: CNN: 95.28%, DFFNN: 100%, LSTM: 83.89%.
Pusuluri et al. [87]	Asphyxia and deaf vs. healthy.	Accuracy, false positive count	Grid search	--	SVM, KNN, RF.	Accuracy: 98.48%.
Farsaie et al. [64]	Central nervous system complications, chromosomal abnormalities, congenital cardiac anomalies, blood disorders.	Average accuracy, recall, specificity, equal error rate, classification error rate.	--	--	SVM, PNN, MLP.	Accuracy: MLP: 91.68%. PNN: 89.93%. SVM: 89.85%.
Onu et al. [88]	Prenatal asphyxia vs. healthy.	Recall, specificity, unweighted average recall.	Random search.	--	ResNet, SVM.	Specificity: 88.9%

## Appendix B

**Table A2:** Definition of different cry segment labels of the database.

Unit	Definition
EXP	Voiced expiratory segment during a period of cry.
EXPN	Unvoiced expiratory segment during a period of cry.
INS	Unvoiced inspiratory segment during a period of cry.
INSV	Voiced inspiratory segment during a period of cry.
EXP2	Voiced expiratory segment during a period of pseudo-cry.
INS2	Voiced inspiratory segment during a period of pseudo-cry.
PSEUDOCRY	Any sound generated by the newborn that is not a cry, such as whimpering.
Speech	Sound of the nurse or parents talking.
Background	A low noise characterized by a low-power silence affected with little noise.
Noisy cry	Any sound heard with a cry (BIP, water, diaper changing, etc.).
Noisy pseudo-cry	Any sound heard with a pseudo-cry.
Noise	Sound originating from the surrounding environment, such as microphone movement, diaper movement, door squeaks, staff chatter, background noise, or speech with BIPs.
BIP	Sound of the medical equipment surrounding the newborn.
